# Culturomics- and metagenomics-based insights into the microbial community and function of rhizosphere soils in Sinai desert farming systems

**DOI:** 10.1186/s40793-023-00463-3

**Published:** 2023-01-13

**Authors:** Wen-Hui Lian, Osama Abdalla Abdelshafy Mohamad, Lei Dong, Ling-Yu Zhang, Dong Wang, Lan Liu, Ming-Xian Han, Shuai Li, Shuang Wang, André Antunes, Bao-Zhu Fang, Jian-Yu Jiao, Wen-Jun Li

**Affiliations:** 1 State Key Laboratory of Biocontrol, Guangdong Provincial Key Laboratory of Plant Resources and Southern Marine Science and Engineering Guangdong Laboratory (Zhuhai), School of Life Sciences, Sun Yat-Sen University, Guangzhou, 510275 People’s Republic of China; 2 Department of Environmental Protection, Faculty of Environmental Agricultural Sciences, Arish University, Arish, 45511 Egypt; 3 College of Life Science and Technology, Honghe University, Mengzi, 661199 People’s Republic of China; 4 Heilongjiang Academy of Black Soil Conservation and Utilization, Harbin, 150086 People’s Republic of China; 5 State Key Laboratory of Lunar and Planetary Sciences, Macau University of Science and Technology (MUST), Macau, People’s Republic of China; 6 State Key Laboratory of Desert and Oasis Ecology, Xinjiang Institute of Ecology and Geography, Chinese Academy of Sciences, Urumqi, 830011 People’s Republic of China

**Keywords:** Culturomics, Metagenomics, Desert farming systems, Rhizosphere microbiome

## Abstract

**Background:**

The microbiome of the Sinai Desert farming system plays an important role in the adaptive strategy of growing crops in a harsh, poly-extreme, desert environment. However, the diversity and function of microbial communities under this unfavorable moisture and nutritional conditions have not yet been investigated. Based on culturomic and metagenomic methods, we analyzed the microbial diversity and function of a total of fourteen rhizosphere soil samples (collected from twelve plants in four farms of the Sinai desert), which may provide a valuable and meaningful guidance for the design of microbial inoculants.

**Results:**

The results revealed a wide range of microbial taxa, including a high proportion of novel undescribed lineages. The composition of the rhizosphere microbial communities differed according to the sampling sites, despite similarities or differences in floristics. Whereas, the functional features of rhizosphere microbiomes were significantly similar in different sampling sites, although the microbial communities and the plant hosts themselves were different. Importantly, microorganisms involved in ecosystem functions are different between the sampling sites, for example nitrogen fixation was prevalent in all sample sites while microorganisms responsible for this process were different.

**Conclusion:**

Here, we provide the first characterization of microbial communities and functions of rhizosphere soil from the Sinai desert farming systems and highlight its unexpectedly high diversity. This study provides evidence that the key microorganisms involved in ecosystem functions are different between sampling sites with different environment conditions, emphasizing the importance of the functional microbiomes of rhizosphere microbial communities. Furthermore, we suggest that microbial inoculants to be used in future agricultural production should select microorganisms that can be involved in plant-microorganism interactions and are already adapted to a similar environmental setting.

**Supplementary Information:**

The online version contains supplementary material available at 10.1186/s40793-023-00463-3.

## Background

As an important part of earth's diverse environments, deserts occupy about one-third of the terrestrial ecosystem. Deserts are extreme environments that are typically dry and host vast communities of unrevealed, unique microorganisms. Previous studies have characterized the diversity and function of microbial communities in different desert environments [1–3], including targeted studies on typical ecological regions within them, such as oases [4] and dry valleys [5]. However, the microbiomes of artificially modified agricultural regions located in the desert (desert farms), remain mysterious [6]. Due to their dry conditions and lack of precipitation, growth of desert farming crops usually depends on continuous water support from irrigation systems, limiting the development of desert farms [6]. Besides that, the impact of microorganisms on desert farms cannot be ignored. Previous studies have emphasized that rhizosphere microbiomes, and their diverse interactions, play an important role in regulating the drought resistance of plants and crops [7–9], and the microbial diversity associated with crops in desert farming is distinct from those in conventional farming systems [10, 11]. Given their exposure to severe drought stress, desert farms are ideal subjects for studies on drought adaptation of crops and rhizosphere microbiomes. Nonetheless, relatively few microbial studies related to desert farms have been reported [11, 12], so it remains unclear whether crops affect soil microbiomes under the extreme nutrient and water limitations of the desert environment.

The diverse interactions between crop and rhizosphere microbiome were exploited in the agricultural system, continuous studies [7–9] have shown that rhizosphere microbiomes play an important role in regulating the stress resistance and growth of plants. Manipulating the rhizosphere microbial community by inoculating microorganisms to enhance stress resistance and improve the yield of crops, which comes with the approach of synthetic microbial communities (SynComs) [13, 14]. The taxonomic and functional information of single strain in the SynComs is clear, so the ecological effects of the entire community and individual strains can be clearly analyzed by the reductionist approach [15]. The SynComs approach has been well practiced in various studies of plant-microbiota interactions, typically in the researches on model plants, such as *Arabidopsis thaliana* [16–18], that have been well studied with clear background of resident microbiota composition. Even though the SynComs inoculation have long been applied in the agriculture systems, like inoculants for biocontrol or biostimulation [19, 20]. However, the filed efficacy of SynComs is inconsistent that varies with different conditions of local environment. It can be explanted by the previous proposed theory of priority effect [21], that is, the establishment of inoculated microorganisms or microbiota in the local microbial community can be mediated by the efforts of the indigenous microbiome, and the effect is usually negative. How to select appropriate microorganisms of specific SynComs to effectively manipulate the rhizosphere microbiome of plants is still an unresolved problem.

In this study, we firstly aimed to determine whether the taxonomic and functional structure of microbial communities inhabiting desert farming system are (1) crop- specific or (2) distinct from those and alternatively linked with the environmental variation brought with change in geographic location. Secondly, and anchored on the previous point, we aimed to provide guidance in improving synthesis of microbial inoculants to assist in future desert agricultural production processes. To this end, we integrated culturomics- and metagenomics-based approaches to provide the first comprehensive view of taxonomic composition and functional trials of the Sinai desert farms rhizosphere microbiome, and also proposed the advice which could be useful in synthesis of microbial inoculants and improvement of current agricultural practices under these settings.

## Materials and methods

### Project design and sampling

The microbial diversity and function of total of fourteen rhizosphere soil samples (collected from twelve plants in four desert farms of the Sinai desert) were investigated in this study (Additional file 1: Table S1, Additional file 2: Figure S1). The rhizosphere soils of cucumber, tomato, lettuce, peas, cabbage, onion, lemon, carob, apricot, mango and guava were sampled from three desert farms (farm A, farm B and farm C). To compare the microbiome of the same plants, we also sampled the rhizosphere soils of one unknown same plant from two desert farms (farm C and farm D). Each sample was obtained from 5 to 10 cm depth near the actively growing roots. Approximately 100 g of soil was collected at each sampling point and kept in sterile plastic bags at air temperature (37 °C) during 3 h transportation to the laboratory in Arish University, Egypt. The samples transported to the laboratory were promptly divided into three parts, one was stored at 4 °C for the isolation and culture of microorganisms; one was placed at room temperature (28 °C) for the physical and chemical determination of the samples; the other was stored in a −80 °C refrigerator for high-throughput sequencing. All samples were tested for determination of total carbon (TC) and total nitrogen (TN) content by using a varioEL C/N analyzer (Elementar Analysensysteme GmbH, Hanau, Germany) (Additional file 1: Table S1).

### Strain isolation and identification

Soil (2.5 g) was suspended in 20 mL sterile distilled water and incubated in a rotary shaker for 1 h (180 rpm, 30 °C). The suspensions were serially diluted and aliquots of 100 μl of each diluted suspension were plated on standard Petri dishes poured with six different freshly prepared growth solid media Reasoner's 2A agar (R2A), Minimal Medium (MM), Trypticase Soy Agar (TSA), Trehalose-Proline medium (TP), mix medium of TSA, R2A and TP (TRT), mix medium of R2A and TP (RT) (Additional file 1: Table S2), supplemented with nalidixic acid (20 mg/L) and nystain (50 mg/L). Preliminary growth experiments were performed with three pH variants (pH 6, pH 7 and pH 8) and plates were incubated at 28 °C, 37 °C and 45 °C under aerobic conditions for two weeks. After comparing the results of the preliminary experiments, plates with pH 7 and incubated at 28 °C were chosen as further culture conditions for isolating the highest number of microbial strains. Single colonies were then tested for purity performing two rounds of streaking on the original isolation media. DNA of each pure culture was extracted using TIANGEN™ and primers 27F-1492R were used for PCR amplification [22]. Amplicons were verified on 0.8% agarose gel with 2 kb DNA ladder (Fermentas) and then sequenced on Sanger platform (Sangon Biotech, Shanghai, China). 16S rRNA gene sequences of the isolates were used for taxonomic identification using RDP classifier [23]. Assigned taxonomies were accepted at estimated confidence higher than 95%.

### 16S rRNA gene amplicon sequencing

Total DNA from each sample was extracted from approximately 20 g of soil by using a DNeasy PowerMax Soil Kit (Qiagen) with an additional step to facilitate cell lysis: after step 2 of the protocol, the PowerMax Bead Tube was frozen at −80 °C for 20 min and then heated at 65 °C for 10 min, with this process being repeated twice; after 10 min, 300 μl lysozyme (50 mg/ml) were added to the tubes which were then incubated in a rotary shaker for 1 h (100 rpm, 37 °C). This was followed by the addition of 150 μl proteinase K (20 mg/ml) and 750 μl SDS (10%), and incubation at 55 °C for 1 h (shaking the tubes every 15 min); 5 ml phenol–chloroform-isoamyl alcohol (25:24:1) were added to the tubes before continuing to step 3 of the DNeasy PowerMax Soil Kit protocol. The extracted DNA was used for 16S rRNA gene amplicon sequencing and shotgun sequencing. The V4 hypervariable region of the 16S rRNA gene was selected for generating amplicons and following taxonomy analysis [24]. Next generation sequencing library preparations and Illumina MiSeq sequencing were conducted at GENEWIZ, Inc. (Suzhou, China).

FastQC was used for checking the quality of fastq files, and 16S rRNA gene sequence analyses were performed with QIIME [25]. The reads were joined and assigned to samples based on barcode, and after demultiplexing and primer removal, joined reads were filtered by mean Phred quality score ≥ 20 and setting a minimum length of 200 bp. Operational taxonomic units (OTUs) were clustered at 97% sequence identity with VSEARCH (v1.9.6) [26]. Taxonomic assignment was performed with RDP classifier [23]. Diversity statistics were performed using QIIME on a rarefied dataset; Alpha diversity was measured with Shannon metrics while Beta diversity was calculated using Bray–Curtis distance matrix and displayed through 2D PCoA plot.

### Shotgun sequencing

Sequencing libraries were prepared following the manufacturer’s protocol (NEBNext® UltraTM DNA Library Prep Kit for Illumina®). The products were cleaned using AxyPrep Mag PCR Clean-up (Axygen), validated using an Agilent 2100 Bioanalyzer (Agilent Technologies, Palo Alto, CA, USA), and quantified by Qubit2.0 Fluorometer (Invitrogen, Carlsbad, CA, USA). Sequencing was performed on Illumina HiSeq platform with 2 × 150 bp paired-ends and an approximate insert size of 350 bp at GENEWIZ, Inc. (Suzhou, China). Adaptors were removed using cutadapt [27] and the reads were quality filtered as described earlier [28]. Microbiome profiling based on taxonomic marker genes was performed using MetaPhlAn2 [29]. The output results were parsed and plotted using GraPhlAn [30].

### Metagenomic data assembly and functional annotation

The high-quality reads of each sample were de novo assembled separately using SPAdes (v3.13.1; -t 30 -m 1000 –meta -k 21,33,55,77,99) [31]. Scaffolds longer than 500 bp were retained and translated to protein-coding open reading frames (ORFs) by using Prodigal [32]. ORFs were annotated using DIAMOND [33] against the KEGG, eggNOG and NR databases by applying E-values < 1e−10. Taxonomic information of each gene was parsed based on NR annotation results. BBMap [34] was used to map high quality reads onto genes, and the script “jgi_summarize_bam_contig_depths” packed in MetaBAT2 [35] was used to calculate the coverage information of each gene. Detailed functional and taxonomic count data can be found in Additional file 1: Table S6 and Table S9.

### Metagenomic binning and data analyses of MAGs

Genome binning was conducted on contigs with a length above 2.5 kbp using MetaBAT2 [35], in which BBMap and “jgi_summarize_bam_contig_depths” with the same parameters were used to compute the coverage of each contigs. Quality control of MAGs was performed by checkM [36] to calculate completeness, contamination and other statistics. Overall, we obtained 867 MAGs classified into medium quality (≥ 50% completeness and < 10% contamination), following the standards of Minimum Information about Metagenome-Assembled Genomes (MIMAG) [37]. The phylogenetic tree was constructed as previous study [38, 39]. Taxonomic assignment of individual MAGs and multiple sequence alignments of conserved proteins were performed using the GTDB-Tk [40, 41]. Final alignments were used to construct phylogenetic trees with IQ-TREE [42] (v1.6.10; -alrt 1000 -bb 1000 –nt AUTO). The best-fit model for Archaea and Bacteria alignments, determined by ModelFinder [43], were LG + F + R4 and LG + F + R10 respectively according to Bayesian Information Criterion (BIC). The relative abundance of the MAGs was estimated based on the recruited reads of each MAG, which was normalized by the total reads. Putative ORFs of all the MAGs were predicted using Prodigal [32] with the “-p single” parameter and annotated as described above.

## Results

### Desert rhizosphere soil microbiome composition remains constant within sampling sites

A combined total of 1,099,402 paired-end raw reads were generated from the 14 rhizosphere soil samples. By quality trimming and sequence filtering, 1,044,045 high-quality reads with an average read length of 292.65 bp were obtained for 16S rRNA gene analysis (Additional file 1: Table S3). A total of 3340 OTUs assigned to 40 distinct phyla were detected in the rhizosphere soil samples (Additional file 1: Table S4).

**Fig. 1:**
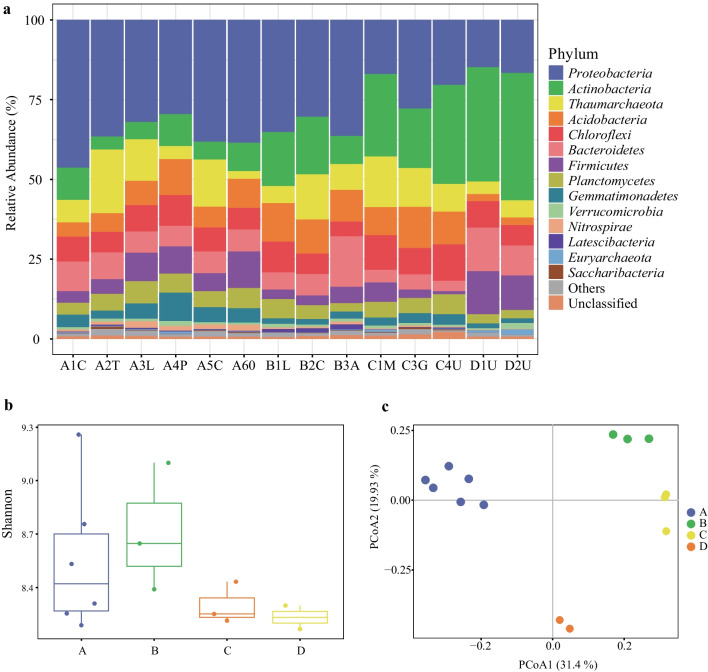
Rhizosphere microbiome composition of desert farm plants inferred by 16S rRNA gene amplicon sequencing. **a** Relative abundance at the phylum level. **b** Shannon alpha diversity index values; Colors represent four sampling sites (A, B, C, D). **c** Principal coordinate analysis (PCoA) using Bray–Curtis distance; Colors represent four sampling sites (A, B, C, D)

The composition of the rhizosphere microbiome (Fig. 1a) was dominated by *Proteobacteria* (29.9% s.d. 9.2%) and *Actinobacteria* (17.1% s.d. 11.4%). Moreover, for the archaea, some phyla were commonly found in all samples, among which *Thaumarchaeota* was the most dominant phylum, and accounted for 9.6% s.d. 5.2% of the rhizosphere microbial community. The comparison of Shannon diversity indices between all sampling sites (Fig. 1b) showed that values from the same sample site were not similar and that sampling sites A and B had generally higher microbial community diversities than C and D. The composition of the rhizosphere microbiome was rather stable among rhizosphere samples collected from the same sample site but displayed obvious differences among sampling sites (Fig. 1c). It is interesting to note that the rhizosphere samples within each sampled site had very similar community compositions, even though their associated plants were quite different.

### Isolates and shotgun metagenomic sequencing cover a wide range of taxa and reveal a high proportion of undescribed taxa

By using multiple different media and conditions, we isolated a total of 528 strains from all rhizosphere samples, which were assigned to 5 phyla, 8 classes and 57 genera (Table 1). Among those isolated strains, most were classified as *Alphaproteobacteria* (298, 56.4%), *Actinobacteria* (69, 13.1%) and *Gammaproteobacteria* (62, 11.7%). Based on the result of RDP classifier (Additional file 1: Table S5), the isolated strains contained a high proportion of potential undescribed taxa (138, 26.1%) ranging from genus to class level.

**Table 1:** Diversity of the strain collection according to the number of taxa isolated at different taxonomic ranks

Sampling site	Number of isolates	Domains	Phyla	Classes	Orders	Families	Genera
A	78	1	4	6	10	15	24
B	125	1	4	6	9	16	24
C	282	1	4	6	13	24	42
D	43	2	4	5	8	13	16
Total	528	2	5	8	15	31	57

Following quality filtering, a total of 1,850,309,601 reads was obtained from the fourteen metagenomic libraries, consisting of 132,164,972 s.d. 18,417,345 reads pairs. After profiling the composition of microbial communities by MetaPhlAn2, the fourteen samples showed obvious difference in taxonomic composition and community structure, especially pronounced in samples from different sites (Additional file 2: Figure S2). The profiling result revealed that *Bacteria* were most abundant (87.7%, 235 taxa), followed by *Archaea* (9.3%, 25 taxa), *Eukarya* (2.6%, 7 taxa) and viruses (0.4%, 1 taxa). A total of 114 taxa out of 268 (42.5%) were identified as unclassified, including unclassified species (100/114, 87.7%) and genera (13/114, 11.4%). Moreover, one unclassified family (belonging to the order *Solirubrobacterales*) was also found. Consistent with the results of the 16S rRNA gene amplicon analysis, *Proteobacteria* (14.9–35.4%) and *Actinobacteria* (6.3–32.9%) dominated the composition of the rhizosphere microbiome in most sampling sites, with *Thaumarchaeota* also being a relevant taxon, yet showing a much wider range of variability in their importance (0.1–75.9%).

**Fig. 2:**
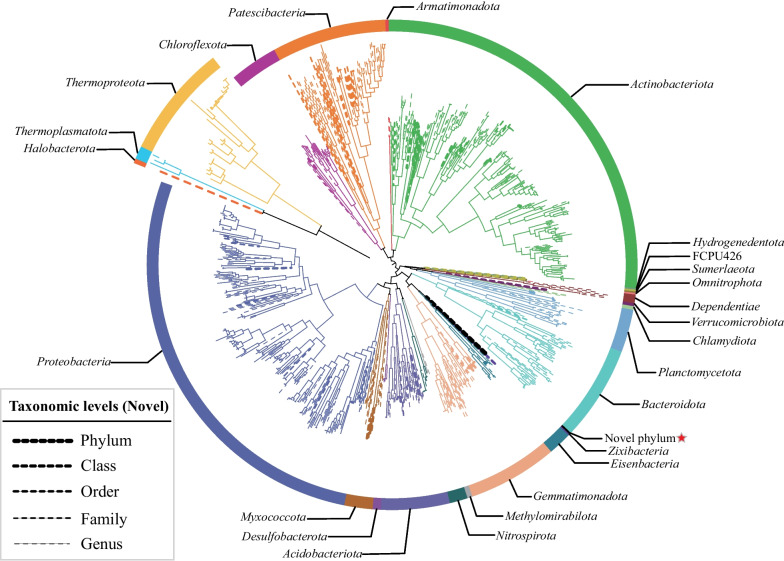
The phylogenetic tree of bacteria and archaea of MAGs based on 120 bacterial marker genes and 122 archaeal marker genes generated from GTDB-tk. Colors of branches and external ring is according to the phylum. Dashed lines of branches represent the potential undescribed microbial taxa, and the width of dashed line shows classification level, from genus to class

To further analyze the taxa in the rhizosphere microbial community, the high-quality reads were assembled and binned for generating metagenome-assembled genomes (MAGs). After quality filtering and assessment of the quality of these genomes, a total of 867 MAGs were obtained with the medium quality (≥ 50% completeness and < 10% contamination); within these, 400 MAGs had a higher quality (≥ 80% completeness and < 5% contamination). The taxonomic assignment of all MAGs (Fig. 2 and Additional file 1: Table S6) inferred by GTDB-Tk showed that most were classified as Bacteria (837/867), and the rest were assigned to the domain Archaea (30/867). In total, MAGs were classified into 22 bacterial phyla and 3 archaeal phyla (Additional file 1: Table S6). The genomes of Bacteria were mainly assigned to *Proteobacteria* and *Actinobacteriota*, while *Thermoproteota* was the dominant taxon for the archaeal MAGs (Fig. 2). Calculating the ANI values between all MAGs and their closest relative genomes available from public database, showed that all MAGs represented undescribed species. Further novelty was detected for higher taxa with undescribed genera (486), families (121), orders (54), classes (2), and phylum (1).

### Metagenomic annotation reveals community functional similarities among four sampling sites, but differ in functional taxa

For profiling the metabolic potential of the rhizosphere microbial community, functional features were inferred from metagenomic datasets using DIAMOND blastp against the KEGG, eggNOG and NR databases. Proteins were classified based on COG categories and further divided into four groups (Additional file 2: Figure S3). It was clearly noticed that the group of METABOLISM constituted the highest proportion of gene abundance, especially COG category E (Amino acid transport and metabolism), followed by COG category C (Energy production and conversion) and G (Carbohydrate transport and metabolism). Notably, the four sampled sites had similar overall community functional composition and abundance (Additional file 1: Table S7). According to gene functional orthologs in KEGG database (Additional file 2: Figure S4), the community functional similarities of four sampling sites were also found, that genes involved in Metabolism, Genetic Information Processing and Environmental Information Processing were both significantly enriched, consistent with observations of functional distribution in the COG categories (Additional file 2: Figure S3). Overall, we can conclude that the rhizosphere microbial communities of the four sampling sites maintain similar ecosystem functions, even though the sites themselves and their plants differ.

**Fig. 3:**
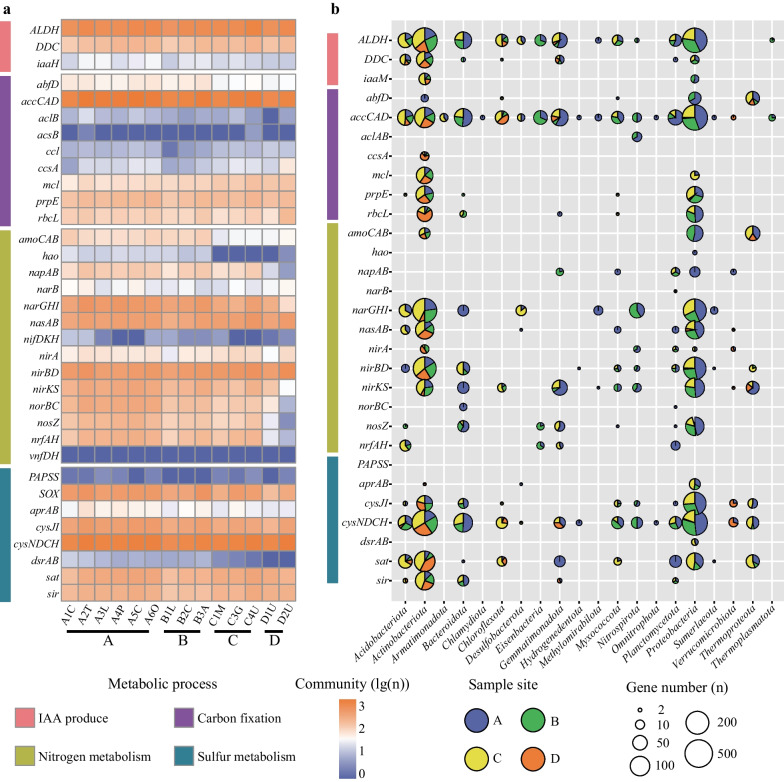
Distribution of metabolic marker genes and microbial taxa involved in specific ecological functional processes within rhizosphere microbial communities sampled from all sampling sites. **a** The abundance of metabolic genes in the metagenomic short reads of each rhizosphere sample, which are normalized by log transformation. **b** The metabolic potential and distribution of the metagenome-assembled genomes (MAGs) in the four sampling sites. Based on the number of rhizosphere samples in each sampling site, percentages of microbial taxa involved in metabolic processes are normalized by averaging

To gain a more detailed overview of the functional profiles of the rhizosphere microbial communities, we analyzed genes encoding key enzymes of the specific ecosystem functional pathways. Considering the high gene abundance linked with metabolism (Additional file 2: Figure S3 and Figure S4), the metabolic pathways associated with plant-rhizosphere microbiome interactions were chosen, including IAA biosynthetic pathway, carbon fixation, nitrogen metabolism and sulfur metabolism (Fig. 3). The links between microbial taxa and ecosystem functions were investigated in different sampling sites by mapping the functional profile against the microbial taxa.

In IAA (indole-3-acetic acid) biosynthesis pathways, the *ALDH* genes (8.2%) that mediate the IPyA pathway was the most abundant in all samples (Fig. 3a and Additional file 1: Table S8), indicating that this pathway is the major IAA biosynthetic pathway in those rhizosphere microbial communities. Moreover, the IAN pathway (*DDC* gene, 1.6%) also showed a relatively important role in IAA production in these communities, but few genes (*iaaM*, *iaaH*) involved in the IAN pathway were annotated (Additional file 1: Table S8). The majority of microbial taxa involved in the IPyA pathway belong to *Actinobacteriota* and *Proteobacteria*, followed by *Bacteroidota*, *Acidobacteriota* and *Gemmatimonadota* (Fig. 3b). However, the DDC genes that mediate the IAN pathway were mainly annotated from *Actinobacteriota* and *Acidobacteriota*, rather than *Proteobacteria* (Fig. 3b and Additional file 1: Table S9). More importantly, the microbial taxa involved in the IPyA pathway were different in the four sampling sites. In sampling sites A and B, *Proteobacteria* was the most dominant phylum involved in the IPyA pathway, while the sampling sites C and D were dominated by *Actinobacteriota*. Notably, members of *Actinobacteriota* were predicted to play an important role in most sampling sites (Fig. 3b and Additional file 1: Table S9).

Five carbon fixation pathways were found in rhizosphere microbial communities, including the reverse tricarboxylic acid cycle (rTCA), the Calvin-Benson-Bassham cycle (CBB), the 3-hydroxypropionate bicycle (3HP-bicycle), the 3-hydroxypropionate/4-hydroxybutyrate cycle (3HP/4HB) and the dicarboxylate/4-hydroxybutyrate cycle (DC/4HB), but a few genes (*acsABCDE/cdhACDE*) involved in the Wood–Ljungdahl pathway were annotated from all metagenomes (Additional file 1: Table S8). The *accCAD* genes (12.5%) involved in the 3HP/4HB pathway showed highest gene abundance in all rhizosphere samples, followed by the 3HP-bicycle pathway (*mcl* and *prpE* genes, 2.5%) and the CBB pathway (*rbcL* gene, 0.9%) (Fig. 3a and Additional file 1: Table S8). Additionally, the *accCAD* genes were commonly annotated in a wide range of microbial taxa, dominated by *Proteobacteria*, *Actinobateriota*, *Bacteroidota*, *Gemmatimonadota* and *Acidobacteriota*. In contrast, the rTCA pathway (*aclAB*/*ccsA* genes) was only found in the *Nitrospirota* and *Actinobacteriota*, respectively, and had a low gene abundance (Fig. 3b and Additional file 1: Table S9). In the CBB pathway (*rbcL* gene), *Actinobacteriota* were dominant in sample site D, while *Proteobacteria* were dominant in sample site A. Moreover, diverse microbial taxa were involved in the 3HP/4HB pathway at sample site A, but other sampling sites only harbored a more restricted taxonomic range. For example, the *accCAD* gene was highly annotated from *Bacteroidota*, *Gemmatimonadota* and *Planctomycetota* in sample site A, while other sampling sites, especially sample site D were dominated by *Actinobacteriota* and *Chloroflexota* (Fig. 3b and Additional file 1: Table S9).

Looking at nitrogen metabolism, several complete metabolic pathways were annotated from the metagenomes, including dissimilatory nitrate reduction (*narGHI*/*napAB* and *nirBD*/*nrfAH* genes), assimilatory nitrate reduction (*narB*/*nasAB* and *nirA* genes) and denitrification (*narGHI*/*napAB*, *nirK*/*nirS*, *norBC* and *nosZ* genes) (Additional file 1: Table S8). The metabolic genes involved in dissimilatory nitrate reduction showed high gene abundance (11.9%) and were dominated by *narGHI* and *nirBD* genes, while few *napAB* and *nrfAH* genes were annotated (Fig. 3a and Additional file 1: Table S8). Additionally, the *narGHI* and *nirBD* genes were present in diverse microbial taxa that differed in all sampled sites. As an examples, in sample site A, the *narGHI* genes were annotated in *Bacteroidota* and *Methylomirabilota* but not in other sampling sites (Fig. 3b and Additional file 1: Table S9). Similarly, a relatively high proportion of predicted genes were involved in assimilatory nitrate reduction (4.8%) and denitrification (10.0%), and differences in functional microbial taxa that contain key genes for those metabolic pathways was found in different sampling sites. Most importantly, genes involved in nitrogen fixation were also annotated, including *nifDKH*/*anfG* and *vnfDKGH* genes (Fig. 3 and Additional file 1: Table S8). However, microorganisms fixating nitrogen were quite different across our rhizosphere samples.

Annotation results of key genes involved in sulfur metabolism, also revealed the active sulfur metabolic activities in these rhizosphere microbial communities. The complete metabolic pathway of assimilatory sulfate reduction was annotated, and the key genes involved in assimilatory sulfate reduction had high gene abundances in all sampling sites, including *cysNDCH*/*sat* and *cysJI*/*sir* genes (Fig. 3a and Additional file 1: Table S8). In contrast, dissimilatory sulfite reductase encoded by *dsrAB* genes showed little abundance in all sampling sites, suggesting a deficiency in dissimilatory sulfite reduction in the rhizosphere microbial communities (Fig. 3a and Additional file 1: Table S8). In assimilatory sulfate reduction, the *sat* gene involved in the reduction of sulfate to sulfite was present in diverse microbial taxa, including *Actinobacteriota*, *Proteobacteria, Acidobacteriota*, and others (Fig. 3b and Additional file 1: Table S9). Notably, the *sat* gene was annotated from different microbial taxa in different sampling sites. For instance, the *sat* gene was derived from *Planctomycetota* and *Gemmatimonadota* in sample site A, which was not the case for other sites (Fig. 3b and Additional file 1: Table S9). In particular, the key genes involved in thiosulfate oxidation by SOX complex were completely annotated with relatively high gene abundance in all sampling sites (Additional file 1: Table S8).

**Fig. 4:**
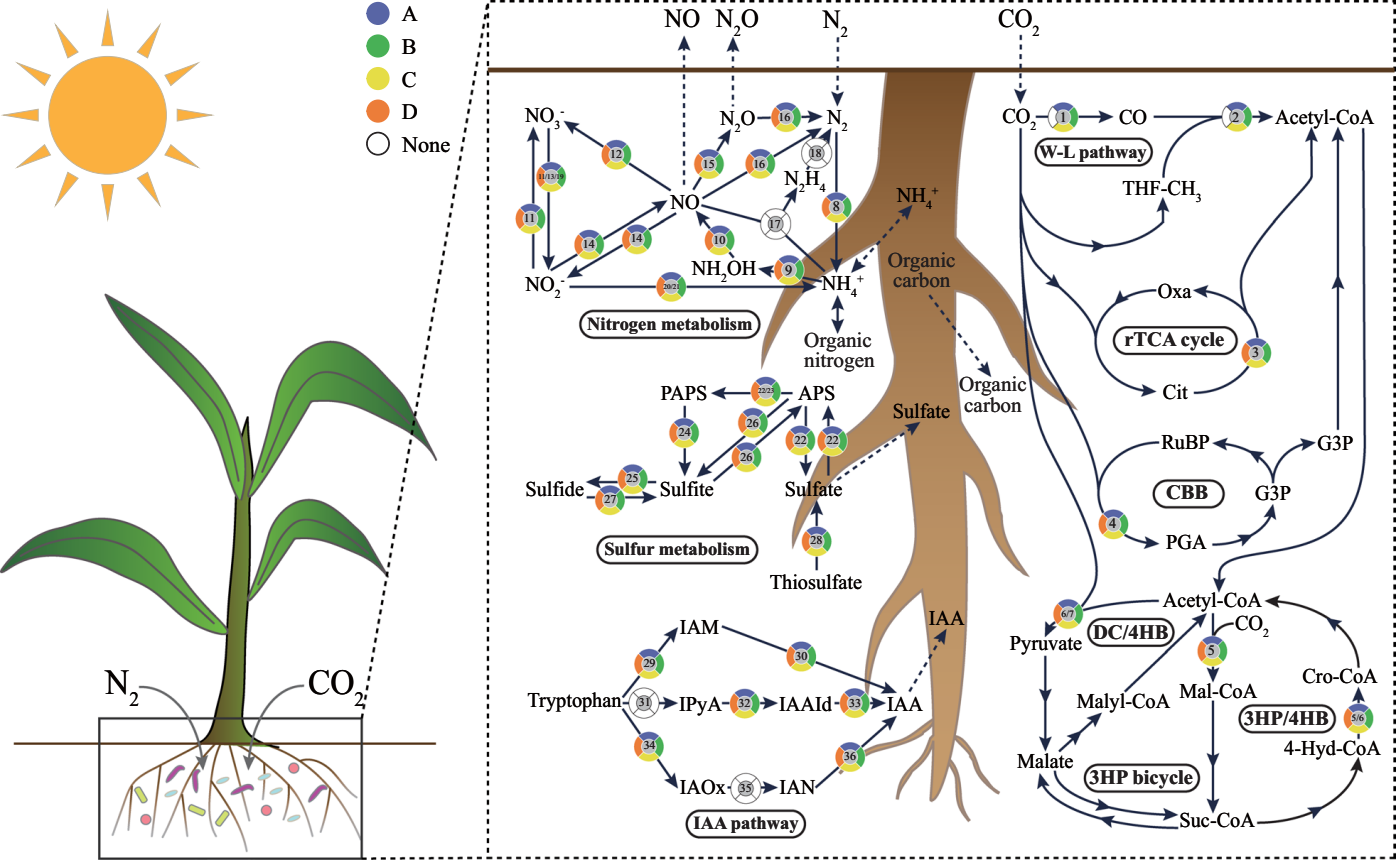
Microbial metabolic activity in the underground of four sampling sites. Colors represent four sampling sites (A, B, C, D). None represents no keys gene of metabolic pathway was annotated in four sampling sites. The numbers in the circle were the numbers of the metabolic pathways in the Additional file 1: Table S8

In summary, the metabolic processes associated with carbon, nitrogen, sulfur and IAA metabolisms were annotated from all rhizosphere samples, with similar relative abundance of key genes involved in the metabolic pathways (Fig. 4), that is consistent with our findings in the overall functional profiles (Additional file 2: Figure S3 and Figure S4). This can be seen, for instance, in the high proportion of predicted genes that were dominant and involved in specific pathways in all rhizosphere samples, including the *ALDH* genes (8.2%) that mediate the IPyA pathway and *accCAD* genes (12.5%) that mediate the 3HP/4HB pathway. It can be concluded that the ecological function composition of rhizosphere microbial communities was similar in different samples (Additional file 2: Figure S3, Figure S4 and Fig. 3a), even when their taxonomic compositions were significantly different (Fig. 1c). By mapping key genes to microbial taxa, the results also suggested that *Actinobacteriota* and *Proteobacteria* might play important roles in local biogeochemical cycles (Fig. 3b and Additional file 1: Table S9). This is further supported by their high relative abundance in rhizosphere microbiome composition (Fig. 1a). In different sampling sites, the dominant phyla that perform the ecosystem functions were different (Fig. 3b). For example, the microbial taxa involved in ecological function of sample site C, D were dominated by *Actinobacteriota*, while the sample site A, B were dominated by *Proteobacteria*. Notably, *Proteobacteria* was predicted to play an important role in all sampling sites, except for the sampling site D, while *Nitrospirota* only showed metabolic potential in sampling sites A and B, and *Verrucomicrobiota* in sampling sites A and C (Fig. 3b).

## Discussion

The diversity of soil microorganisms is an important indicator of soil quality [44] and plant health [45], which is also essential for the maintenance of soil ecosystem functions. In this study, we integrated culture-dependent and -independent methods to investigate rhizosphere microbial diversity at our target environment, leading to the isolation of 528 pure strains and assembly of 837 MAGs, covering a wide diversity of microbial taxa. Based on our results, both *Proteobacteria* and *Actinobacteria* showed high abundance, which is consistent with prior studies in other arid and semi-arid environments [46, 47]. Interestingly, members of the phylum *Thaumarchaeota* (GTDB: c_Nitrososphaeria) -the dominant group within the archaeal community- were present in relatively high proportions. This might be related to the mesophilic and thermophilic adaptability of *Thaumarchaeota*, or their ability to oxidize ammonia aerobically [48]. In addition to the aforementioned three phyla, other phyla are found to be enriched in the samples, including *Acidobacteria*, *Chloroflexi*, *Bacteroidetes*, *Firmicutes* and *Gemmatimonadetes*. Fittingly, previous studies suggested that these phyla might be indispensable for maintaining ecosystem functions and nutrient cycling in desert environment, namely in carbon and nitrogen fixation [49]. The isolated strains and MAGs contained a high proportion of undescribed taxa, many of which were also involved in important soil functions, indicating a vast richness of untapped microbial resources in the desert farming system.

Previous studies revealed the dynamics of soil microbial community structure response to the state of the soil environment, such as soil drought [7, 9], acidification [50], or diseases [51]. In this study, the microbial community structure of rhizosphere samples within each sampling site were more similar to each other, even when their plant hosts were quite different, indicating that soil properties dominated the assembly of the rhizosphere microbial community. Although some previous studies had shown that soil microbial community structure is usually related to plant type and agricultural management [52], this seems not to be the case here. Indeed, the possible dominant factors at play in the current study are linked with soil properties rather than plant type. This has also been previously proposed by other authors in a variety of environments, including farmland [53, 54], desert [55], tropical seagrass beds [56], etc.

In the current study, we found similar functional composition and abundance in different sampling sites, despite significant differences in the taxonomic composition of their rhizosphere microbial communities. The biogeography of soil microorganisms, particularly those carrying out soil metabolic processes is linked with the ecological function traits of soils [57]. Furthermore, by mapping the soil microorganisms to the metabolic pathways associated with plant-rhizosphere microbiome interactions, we observed that the abundances of key genes related to specific metabolic processes were similar across sampling sites, but the microorganisms involved in these metabolic processes were obviously different. In summary, our results emphasize the importance of the functional microbiomes of rhizosphere microbial communities.

**Fig. 5:**
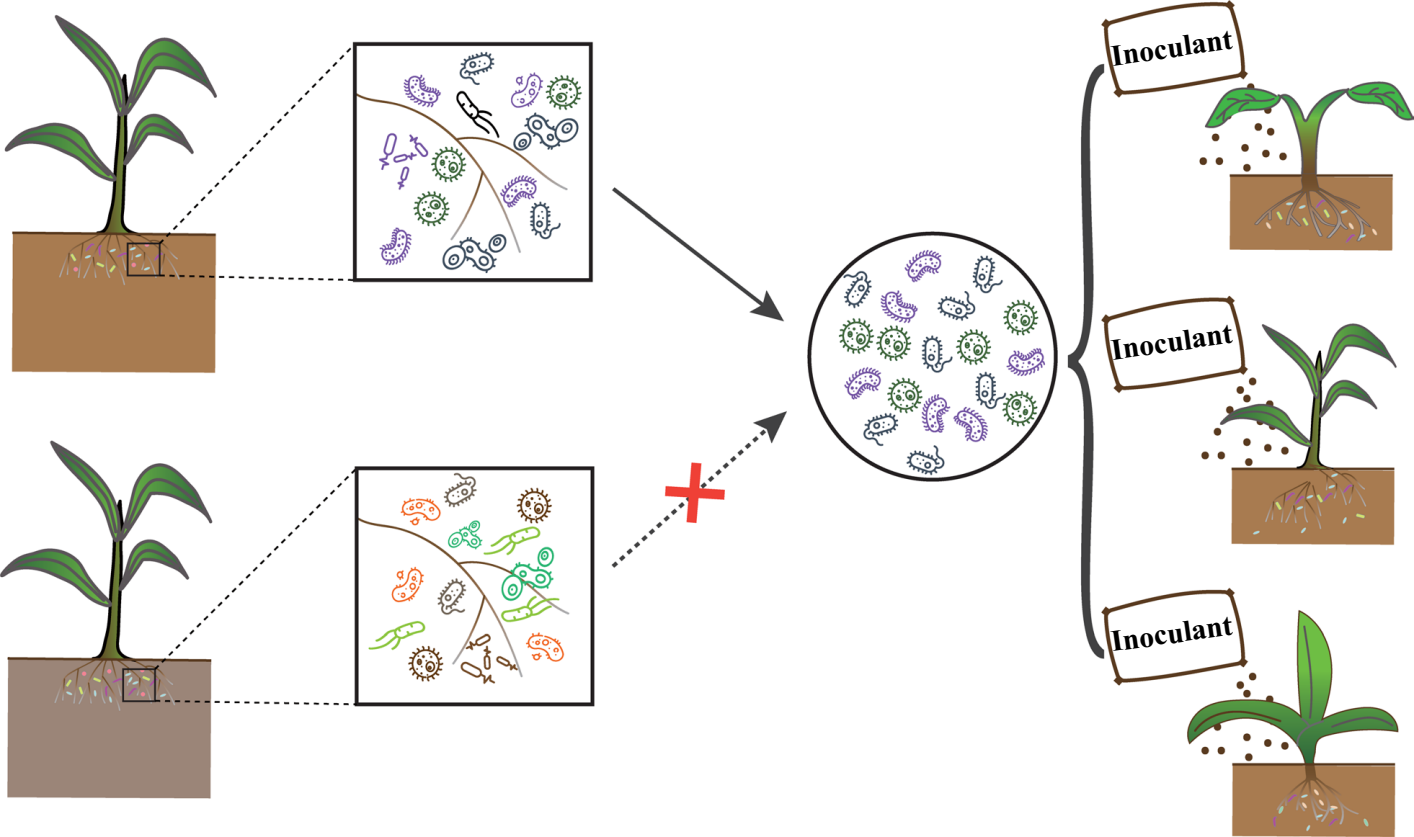
Proposed strategy for the design of microbial inoculants. The composition of rhizosphere microbial community is different among the different soil conditions of rhizosphere environments. The functional microbiome of rhizosphere microbial communities under different soil conditions must be taken into consideration, selecting the soil microorganisms that carry out metabolic functions and adapted to local soil environments

Based on these results, we propose a new approach in the design of microbial inoculants which takes into account that different soil properties generate different microbiomes (diversity and function etc.). According to our preliminary results, the rhizosphere shapes the same ecological functions with different microbial communities, emphasizing the importance of the functional microbiome rather than the taxonomic microbiome. In the design of microbial inoculants, one usually considers the metabolic function and interactions of microorganisms in these communities at the individual level [13]. Only rarely are the functional microbiomes of rhizosphere microbial communities under real soil conditions systematically considered. This results in inconsistent field efficacy of microbial inoculants, varying with the specific conditions of the different environments where they are being applied [45]. To address this critical problem, the functional microbiomes of rhizosphere microbial communities under different soil conditions must be taken into consideration. This would allow for selection of microorganisms that carry out key metabolic functions and are also already adapted to the local soil environments. As shown in Fig. 5, in the rhizosphere environment, the composition of rhizosphere microbial community is different under different soil conditions. Based on our results, one could target the isolation of these key functional microbial taxa to construct soil-associated inoculants and then use them to inoculate local farm soils with similar soil properties. Using this approach, the soil-associated inoculants can more easily adapt to the local soil environment and play a more effective role in diverse plant growth regulation and increased crop yields.

## Conclusions

A deep understanding of microbial diversity and function of rhizosphere microbiomes could further provide the comprehensive and directional insights required for manipulating these microbial communities. Oriented by this idea, our study provides the first comprehensive insights into microbial diversity and function of rhizosphere microbiomes in the Sinai desert farming systems. Our results revealed that variations in rhizosphere microbiome composition were associated with changes in geographic location our sampling, rather than with the type of crop. These rhizosphere microbial communities included a diverse composition but stable metabolic function. Importantly, key microorganisms involved in ecosystem functions which are different between the sampling sites, indicating the "soil-bacteria veins". Based on the results of this study, we suggest that microbial inoculants to be used in future agricultural production should selecting microorganisms that can be involved in plant-microorganism interactions and are already adapted to a similar environmental setting. This principle should be validated and used to guide the targeted synthesis of improved microbial inoculants to assist in future agricultural production processes in desert farms and beyond. Our preliminary results will support further work required to confirm the generality of these results in field experiments and other farming systems and on its potential for implementation.

## Supplementary Information

**Additional file 1:** **Table S1:** Description and physicochemical characteristics of the samples. **Table S2:** The composition and dosage information of culture media. **Table S3:** The sequencing quality of reads for each rhizosphere sample. **Table S4:** OTU table with taxonomic information for each rhizosphere sample. **Table S5:** The taxonomic information of isolated strains by RDP classifier based on 16S rRNA gene sequences. **Table S6:** The GTDB taxonomy and CheckM results of 867 MAGs. **Table S7:** The metabolic functional annotation information of all rhizosphere samples based on COG database. **Table S8:** The key genes of four metabolic functions in the four sampling sites. **Table S9:** The key genes of four metabolic functions in 400 MAGs.

**Additional file 2:** **Figure S1:** The analysis pipeline of the culturomic and metagenomic methods in microbial community and functional of rhizosphere soils in Sinai desert farming systems. **Figure S2:** Taxonomic tree of taxa detected in all rhizosphere samples by shotgun metagenomic sequencing. The external rings represent microbiome composition at each rhizosphere sample, with maximum color intensity corresponding to a relative abundance > 1%. Colors of branches represent Top 10 phyla (or classes for *Proteobacteria*), which uncolored branches represent other phyla. Shapes of the leaves of branches represent as follows: dots, described taxa; stars, unclassified genera; diamonds, unclassified families. Shape size represents the number of microbial taxa. **Figure S3:** Percentages of proteins annotated within each rhizosphere sample for all COG categories. The functional features of rhizosphere microbiomes in four sampling sites showed no significant difference in all COG categories. The COG categories are divided into four major groups, INFORMATION STORAGE AND PROCESSING, CELLULAR PROCESSES AND SIGNALING, METABOLISM, POORLY CHARACTERIZED. Colors represent four sampling sites (A, B, C, D). **Figure S4:** Heatmap showing metabolic pathways of the rhizosphere microbial communities in each sample at two levels of KEGG Orthology.

## Data Availability

The 16S rRNA gene amplicon and metagenome data are available in NCBI Sequence Read Archive (SRA) database under the project PRJNA810924 (SRA accession numbers from SRR18163165 to SRR18163178) and PRJNA810925 (SRA accession numbers from SRR18218230 to SRR18218243). The 16S rRNA gene sequences of all isolated strains are deposited in the NCBI GenBank database with the accession numbers from OM884476 to OM884998.
