# Supplementary material for: Culturomics- and metagenomics-based insights into the microbial community and function of rhizosphere soils in Sinai desert farming systems
Source: Environ Microbiome. 2023 Jan 13;18:4. doi: 10.1186/s40793-023-00463-3 (PMC9840269; doi:10.1186/s40793-023-00463-3)
Supplement: Supplementary file 2 — Additional file 2: Figure S1: The analysis pipeline of the culturomic and metagenomic methods in microbial community and functional of rhizosphere soils in Sinai desert farming systems. Figure S2: Taxonomic tree of taxa detected in all rhizosphere samples by shotgun metagenomic sequencing. The external rings represent microbiome composition at each rhizosphere sample, with maximum color intensity corresponding to a relative abundance > 1%. Colors of branches represent Top 10 phyla (or classes for Proteobacteria), which uncolored branches represent other phyla. Shapes of the leaves of branches represent as follows: dots, described taxa; stars, unclassified genera; diamonds, unclassified families. Shape size represents the number of microbial taxa. Figure S3: Percentages of proteins annotated within each rhizosphere sample for all COG categories. The functional features of rhizosphere microbiomes in four sampling sites showed no significant difference in all COG categories. The COG categories are divided into four major groups, INFORMATION STORAGE AND PROCESSING, CELLULAR PROCESSES AND SIGNALING, METABOLISM, POORLY CHARACTERIZED. Colors represent four sampling sites (A, B, C, D). Figure S4: Heatmap showing metabolic pathways of the rhizosphere microbial communities in each sample at two levels of KEGG Orthology. [file 40793_2023_463_MOESM2_ESM.pdf]

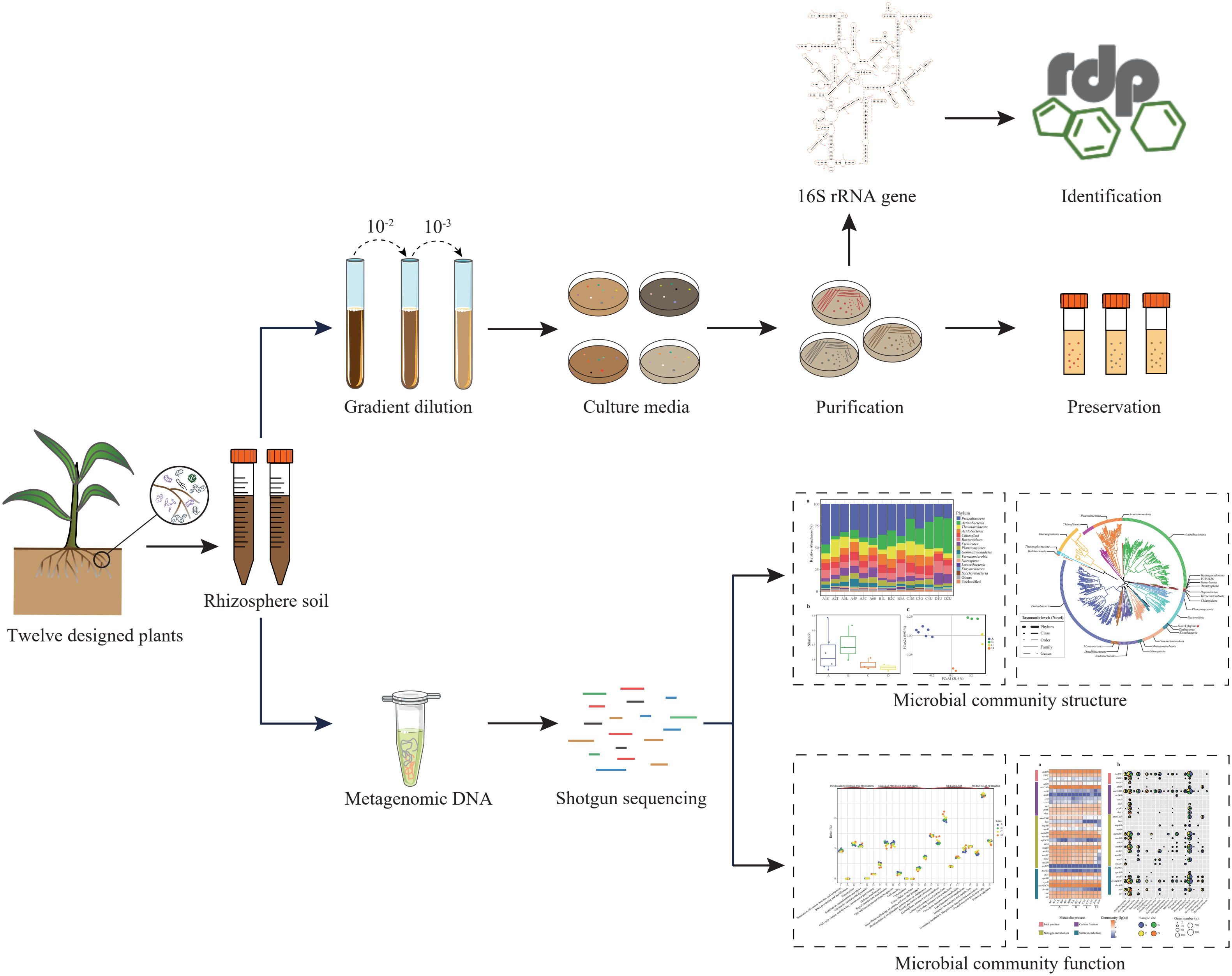

Figure S1. The analysis pipeline of the culturomic and metagenomic methods in microbial community and functional of rhizosphere soils in Sinai desert farming systems.

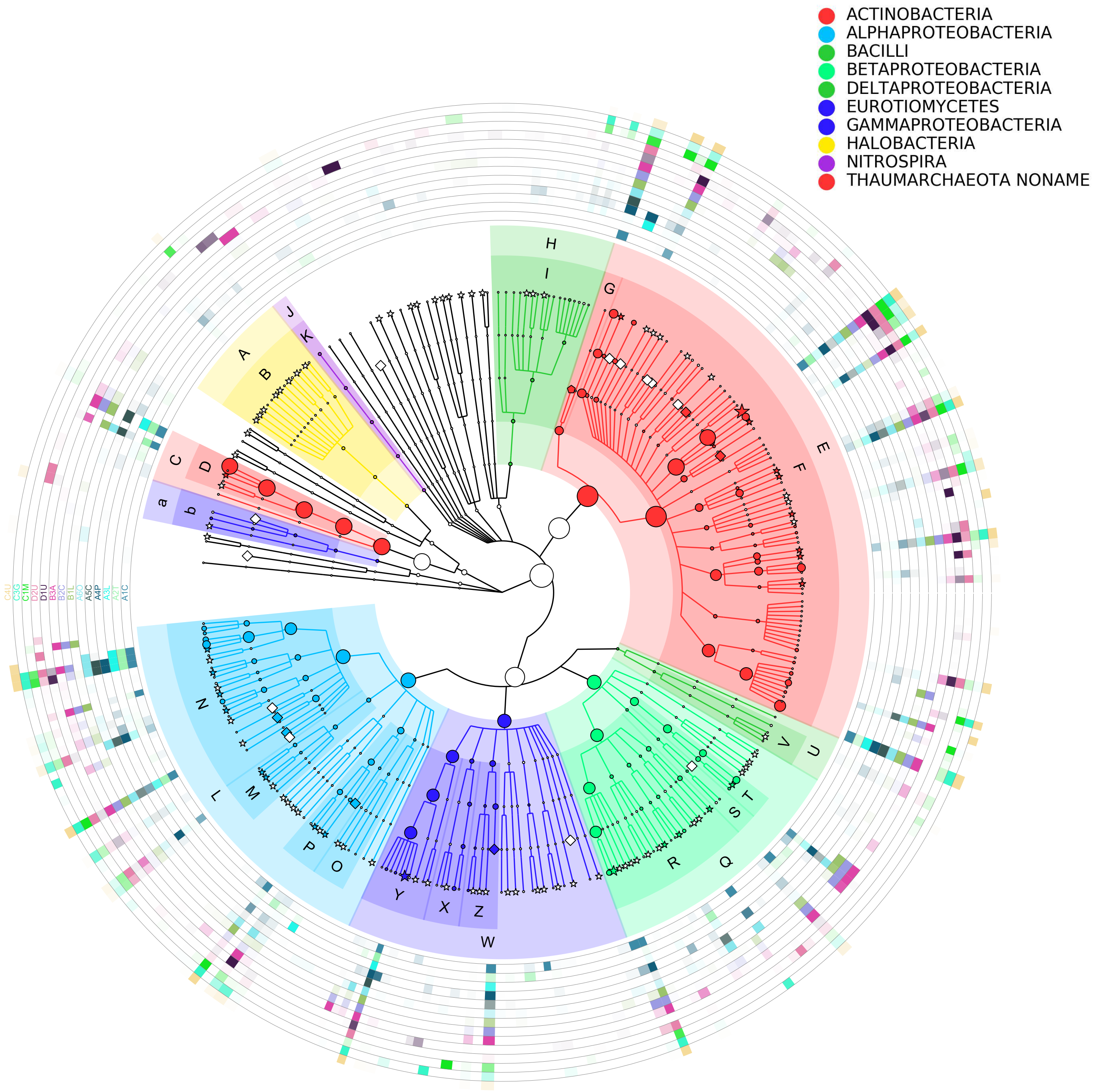

Figure S2. Taxonomic tree of taxa detected in all rhizosphere samples by shotgun metagenomic sequencing. The external rings represent microbiome composition at each rhizosphere sample, with maximum color intensity corresponding to a relative abundance > 1%. Colors of branches represent Top 10 phyla (or classes for Proteobacteria), which uncolored branches represent other phyla. Shapes of the leaves of branches represent as follows: dots, described taxa; stars, unclassified genera; diamonds, unclassified families. Shape size represents the number of microbial taxa.

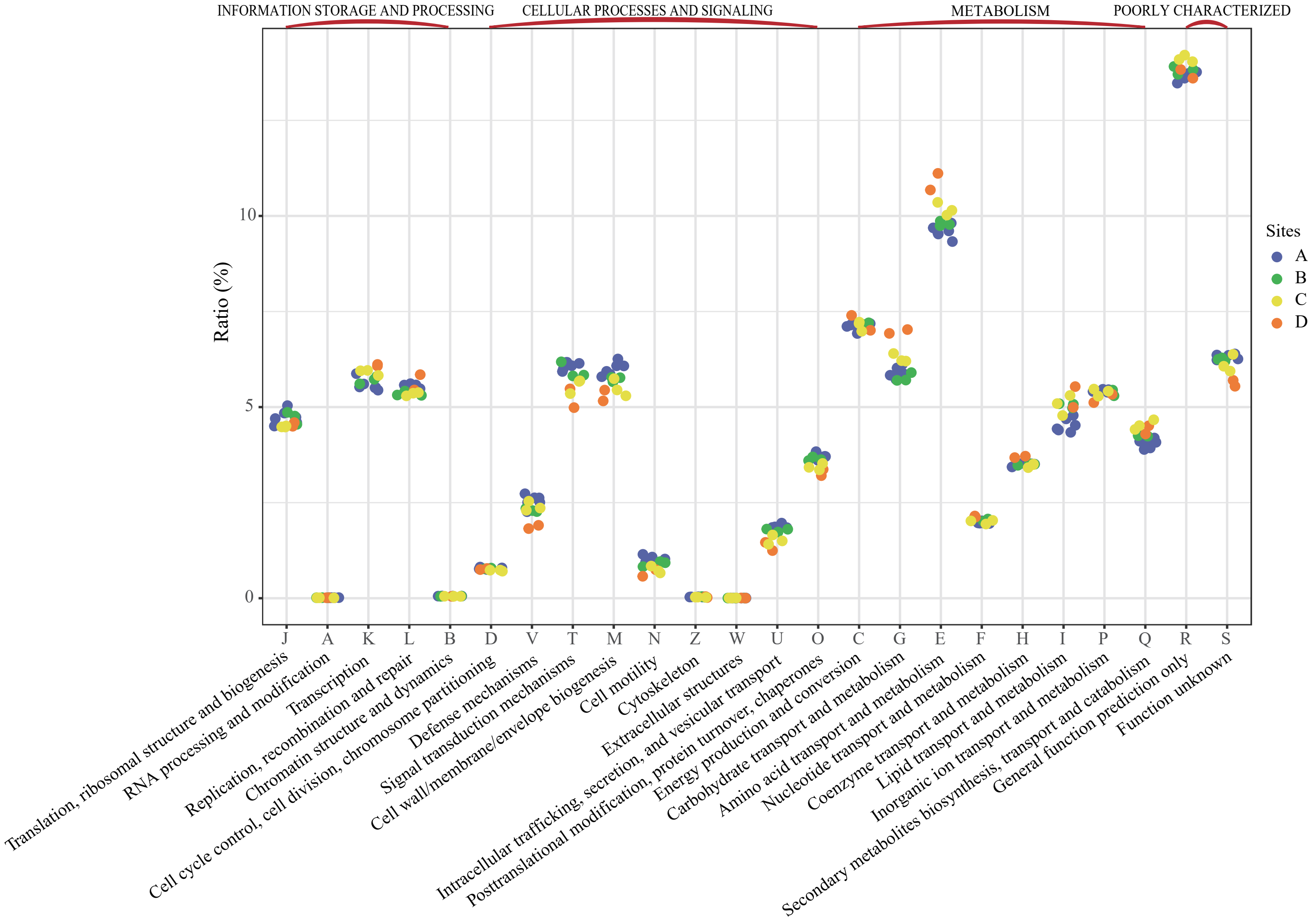

Figure S3. Percentages of proteins annotated within each rhizosphere sample for all COG categories. The functional features of rhizosphere microbiomes in four sampling sites showed no significant difference in all COG categories. The COG categories are divided into four major groups, INFORMATION STORAGE AND PROCESSING, CELLULAR PROCESSES AND SIGNALING, METABOLISM, POORLY CHARACTERIZED. Colors represent four sampling sites (A, B, C, D).

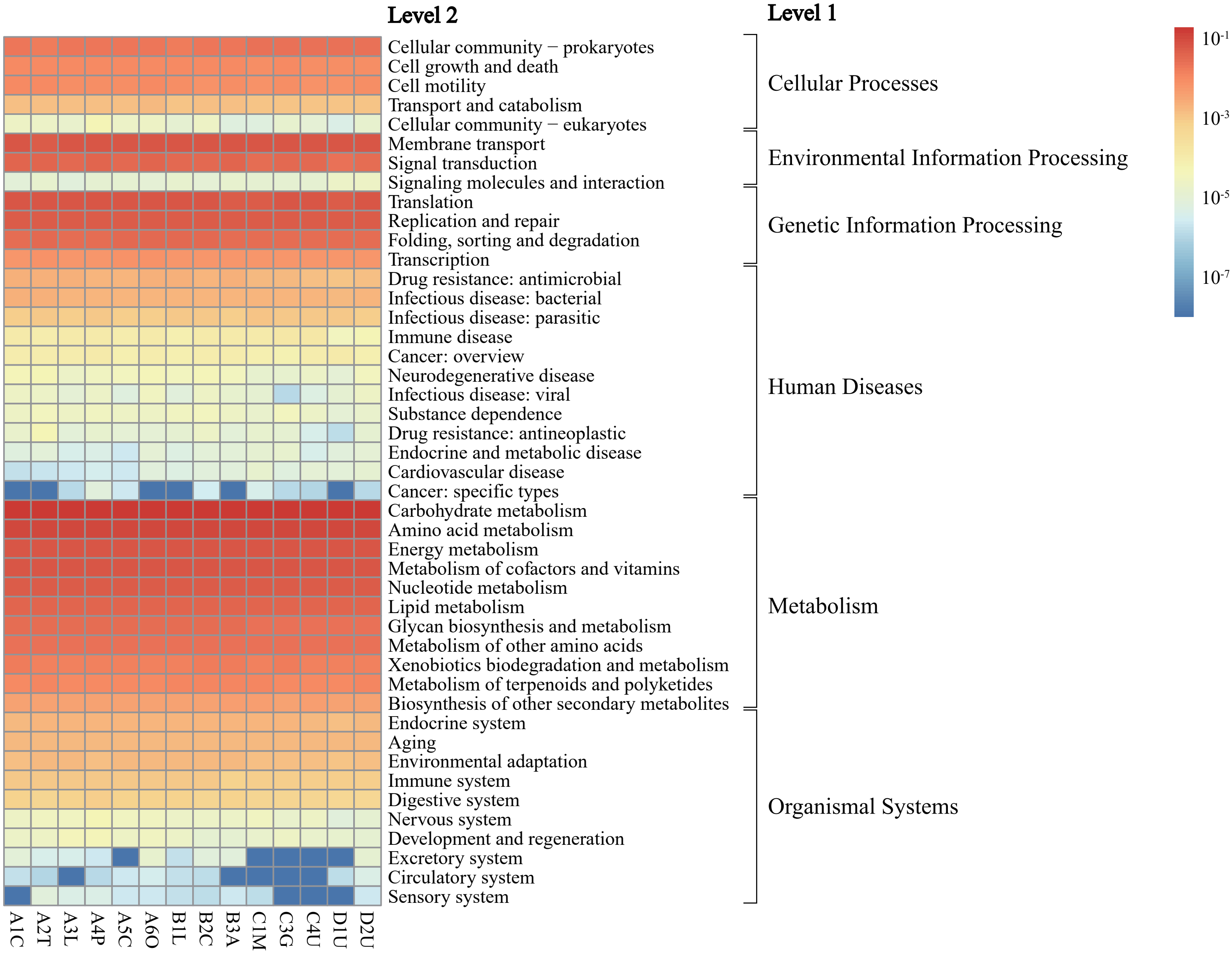

Figure S4. Heatmap showing metabolic pathways of the rhizosphere microbial communities in each sample at two levels of KEGG Orthology.
